# Measuring workload for tuberculosis service provision at primary care level: a methodology

**DOI:** 10.1186/1478-4491-10-11

**Published:** 2012-05-28

**Authors:** Lucie Blok, Susan van den Hof, Sayoki G Mfinanga, Amos Kahwa, Esther Ngadaya, Liesbeth Oey, Marjolein Dieleman

**Affiliations:** 1 Development Policy and Practice, Royal Tropical Institute (KIT), P.O. Box 95001, Amsterdam 1090 HA, The Netherlands; 2 Research unit, KNCV Tuberculosis Foundation, P.O. Box 146, The Hague 2501 CC, The Netherlands; 3 Department of Global Health, Academic Medical Center and Amsterdam Institute of Global Health and Development, P.O. Box 22700, Amsterdam 1100 DE, The Netherlands; 4 Muhimbili Medical Research Centre, National Institute for Medical Research (NIMR), P.O. BOX 3436, Dar es Salaam, Tanzania; 5 Unit Africa, KNCV Tuberculosis Foundation, P.O. Box 146, The Hague 2501 CC, The Netherlands

## Abstract

We developed and piloted a methodology to establish TB related work load at primary care level for clinical and laboratory staff. Workload is influenced by activities to be implemented, time to perform them, their frequency and patient load. Of particular importance is the patient pathway for diagnosis and treatment and the frequency of clinic visits. Using observation with checklists, clocking, interviews and review of registers, allows assessing the contribution of different factors on the workload.

## Background

In view of reaching the Millennium Development Goals' (MDG) targets for health, the demand for well trained and productive health staff in global health programmes is high [1]. The global Stop TB Strategic Plan 2006-2015, established to facilitate the achievement of TB-related MDGs, describes the need for expanded services in order to halt and reverse TB incidence [2]. The application of new technologies and improvements in service delivery according to quality standards, create additional demands on health staff. Therefore, the global strategic plan for TB stresses the importance of careful planning for human resources for health, citing the insufficient quantity and quality and the mal-distribution of staff as the main barriers limiting effective TB control [3].

Given the current crisis in human resources for health in many resource-poor countries, combined with often major staff shortages in rural and remote areas, rational planning for HRH is crucial and requires better justification of staffing requests for specific interventions. The use of generic staffing norms for planning of human resources for health (HRH) in TB control is unsatisfactory. Field experience shows that there is significant variability in workload and productivity of staff within and between countries [4,5]. Major factors contributing to this are patient load, organisation of services and Human Resource Management activities, all will be different in different settings. This implies that context specific staffing norms need to be developed to maximize effectiveness of services, and thereby assuring to reach the TB program targets. Such norms can only be formulated on the basis of local evidence. In light of this, a comprehensive insight into the organization of services, the patient flow in TB control, and the workload of staff within countries or districts is required to estimate HRH requirements.

In this article we describe a methodology that we developed to establish TB related work load in a given context and for a given patient load for use by TB program managers and health planners. We piloted this methodology in Tanzania in three districts; one rural, one urban and one semi-urban district. Tanzania is an appropriate location to pilot the study as the country currently faces a health workforce shortage of 65% in the public sector and 86% in the private sector, with unequal distribution between urban and rural areas [6].

Workload is defined in this article as "the total time required to provide all TB services for a certain patient load in a defined period of time" (adapted from Needham [7]).

The scope of our study in Tanzania is limited to evaluating workload regarding patient and community service delivery tasks at primary care level. It is especially at this level that shortages of qualified staff occur and where most TB patients are diagnosed and (should) receive care. In this article we present and discuss the methodology to measure actual workload for TB-related tasks at primary care level.

## Methodology

### Review of available methods

The first step was a comprehensive review of published and grey literature on work load study methodologies and other methods to determine staffing requirements. Analysis of a total of 25 studies, models or tools that fulfilled the review criteria, shows that over the past years, a number of methodologies have been developed to measure or estimate HR requirements for various health services [8]. Generally, two types of focus were found amongst the methods described. On the one hand, there are studies that aimed to assess the current workload status of staff within a specific context [9-14]. In many cases this was related to a broader purpose of the study such as assessing the effect of organizational changes [15,16] to explore workload related to other staff dynamics [17-19], to quantify the state of health service delivery nationwide [20] or to improve quality or efficiency of the services [21,22].

The second type of methodologies included 11 studies and models that principally aimed at quantifying staff requirements to provide certain services. Five of these [23-27] used the Workload Indicators for Staffing Needs (WISN) methodology developed by the WHO [28]. In this method, HR estimations are calculated by appraising the tasks that are needed to provide the services followed by estimation of the time required per task. Other studies, the Zambian workforce study [29] and the Quantity Task Productivity model [5] emphasized more on productivity. Four HR estimation methods were based on projections [30-33]. Among the reviewed documents we found a variety in scope of the studies and specificity of the workload calculations. Different approaches and methodologies are used for data collection such as various forms of direct observation, self-reported data, or review of secondary data. The review showed that none of the available methodologies would be adequate to calculate workload for TB, although certain elements of these methodologies can be used.

### Protocol development and piloting

A protocol to assess workload of TB services at primary care level was developed and tested as a collaborative effort between KIT, KNCV Tuberculosis Foundation, the National Institute for Medical Research, Muhimbili Medical Research Centre (NIMR-Muhimbili), and the National TB and Leprosy control Program (NTLP) of Tanzania. After training of the researchers, initial field testing and adaptation of the draft generic protocol to the specific Tanzanian context the methodology was piloted in three districts of Tanzania. The results of the pilot were used to finalise the methodology presented below.

### Scope, concepts and variables

The workload components evaluated in our study include all tasks in TB diagnosis and care, all registration, supervision and training-related time spent by service delivery staff and all tasks related to advocacy, communication and social mobilization which are implemented by primary care service providers. With regard to staffing cadres, all staff at primary care service delivery level performing TB related tasks, including lab staff, is covered. Supervisory and district management tasks are excluded.

**Figure 1:**
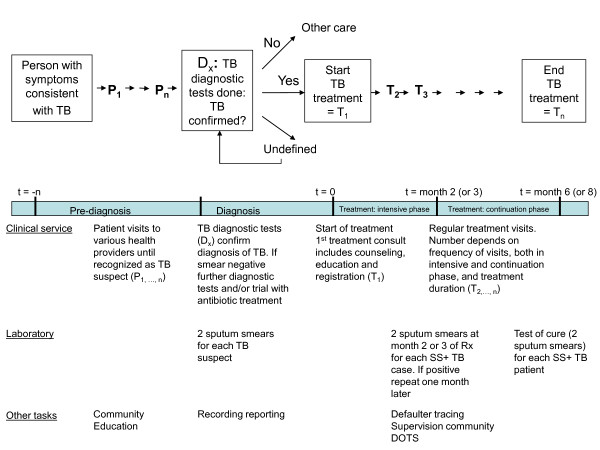
**Patient Pathway in TB Diagnosis and Treatment**.

Workload by primary care level cadres regarding implementing TB services and activities at primary care level is considered to be composed of a number of components:

The *first *element is the time required to implement all the different tasks needed to provide a TB service according to standard quality, which is defined in this study as "performing tasks according to algorithms, guidelines or protocols" and is visualised in Figure 1. The time required is influenced by the diagnostic and treatment protocols chosen, the organization of services and patient flow, the frequency and numbers of follow up visits and the experience and expertise of the staff.

The *second *element is the TB case load. The number of cases diagnosed and treated depends on disease burden and is further influenced by patient's health seeking behaviour, access to services and by the responsiveness and ability of health providers to identify TB suspects. The *third *element of TB workload is the efficiency of the system within which the staff operates. This is influenced by the distribution of cases and services and adequate management of resources.

Furthermore, the required number of staff is also influenced by staff productivity, which is highly context-specific and is influenced by training and staff motivation and depends on the organization of services as well as adequate availability and use of resources [34,35]. The study protocol developed by our team uses mixed methods and covers all of these elements. In this article however we focus on the description of the methodology to measure actual workload.

### Pilot study

We developed a methodology to assess the actual workload related to TB tasks at patient service delivery level within a specific context. We piloted this methodology with data collection focussing on:

i. The programme context, including TB and HIV/AIDS epidemiology; structure of the NTLP; organization of TB service delivery; prevention, diagnosis, treatment and care at the health facility and community level; program results;

ii. Assessment of actual workload, that was operationalized as the average actual measured time for each service delivery task, the observed average frequency of these different service delivery tasks for each patient and the estimated average time for TB program support tasks, in combination with patient load.

iii. Reviewing how the time used to perform TB related tasks correlates with the quality and completeness of task performance against standard protocol.

We used the information to calculate how much time is needed to provide services for one TB patient from the moment (s) he is seeking care up to the moment a patient is declared cured. We calculated this for clinical and lab services separately and combined. In Tanzania, TB services are integrated with other general services in certain facilities, but in places where the patient load is high, TB specific staff may be appointed. This means that the percentage of time dedicated to TB services per cadre will vary per facility. For this reason we have calculated the time required per patient, allowing managers to asses in their own facility what this implies for staffing requirements. No distinction in workload calculations is made between doctors, nurses, clinical officers, etc., as in facilities different types of primary care providers may be responsible for TB tasks.

## Results

Here we first describe the data collection methods used to measure workload for all steps in the patient pathway. This is followed by the actual calculation of workload in three districts in Tanzania. We will only mention differences between districts when they are statistically significant.

### Data collection methods

The methodology developed uses mixed methods and includes observations and clocking of specific tasks, compilation of standardised data from TB registers and reports, structured interviews with patients and health staff and semi-structured interviews. Given the different steps in the patient pathway (see Figure 1) workload data are compiled for the pre-diagnosis period, for the intensive treatment phase and for the continuation treatment phase and calculated separately for the clinical service providers and the laboratory staff.

#### Clinical services

Patient provider encounters were observed and the duration of different types of consultations was clocked using a stop watch. Check lists were used during the observations of diagnostic and treatment consultations to establish completeness with regards to the various tasks according to the standard TB protocols. The checklists were developed on the basis of a task analysis in management of TB and on existing standard operating procedures for TB control [36] and adapted to the organisation of services in Tanzania.

TB patients' diagnostic pathways, including the average number of patient-provider contacts, beginning as a TB suspect continuing until completion of treatment, were assessed through structured interviews with TB patients at facility level and semi-structured and structured interviews with the TB supervisor. TB statistics, such as treatment outcomes, and clinical records were used to assess the caseload and program performance at district level.

#### Laboratory

The research team measured the time required for sputum smears by clocking the time it took to examine sputum smears (including time to instruct patients, receive sputum, prepare and read smears and register and report results) and through interviews with laboratory technicians. Instruments used for observations of slide reading were a checklist for lab staff and a stopwatch.

#### Other tasks

Lastly, we established the time spent on indirect TB-related activities. Indirect TB tasks include administrative tasks such as recording and reporting, TB drugs stock management and ordering or quality assurance. It further consists of time related to receiving supervision, attending TB related trainings and meetings as well as time spent on community outreach and defaulter tracing. The total amount of time spent on these activities during a year was estimated for each TB facility based on structured interviews with the person in charge of the TB activities and was inventoried separately for laboratory personnel and other health staff providing TB services.

## Measuring workload of TB-related tasks

### Clinical service workload for diagnosis of TB

The average number of patient provider contacts required before a diagnosis is reached was established through interviews with patients currently on treatment. The number of visits required depends on the capacity of a general health provider to recognize TB related symptoms and on the way the services are organised. Patients reported that they had undergone an average of 2.0 diagnostic consultations of any formal health provider before their diagnosis was confirmed. The average number of pre-diagnostic visits was higher in the rural district (3.0 visits) than in the two (semi-) urban districts (1.8 visits). The reason for this difference is not clear but relative inexperience of rural health workers and the distance to diagnostic facilities may have contributed to delayed referral for sputum check. We also found a slight difference in number of consultations before diagnosis by type of TB varying from 1.8 for smear negative pulmonary TB to 2.4 for extra pulmonary TB, however these differences were considered too small to have a major impact on the workload calculations.

The time spent on each patient during a diagnostic or treatment consultation was measured by clocking the total duration of the consultation and noting the number of seconds. A distinction was made between patients with symptoms consistent with TB in various stages of diagnosis and patients with other types of symptoms.

There was a significant difference in average duration between a patient coming with TB like symptoms with a mean of 7 minutes 9 seconds (median 5 minutes 24 seconds, n = 38) and patients with other symptoms (mean 4 minutes 6 seconds; median 3 minutes 18 seconds; n = 776). Despite a possible Hawthorn effect or observation bias, none of the consultations was complete with regards to history taking and physical examination. To observe quality and completeness of each consultation the researchers recorded on a check list which elements of the standard consultation protocol were covered, such as specific questions to be asked during history taking and elements to be covered in physical examination. In all consultations elements were missing, therefore we conclude that the measured average time of 7 minutes 9 seconds is an underestimation of the required time for a complete and good quality consultation. For example a separate analysis of consultations in which at least 3 out of 6 questions were asked and some form of physical exam performed and the suspected diagnosis of TB was explained, resulted in an average duration of 13 minutes 16 seconds (median 13 minutes 12 seconds, n = 4). If at least one of these elements were included the average time was 10 minutes 12 seconds (median 10 minutes 2 seconds, n = 25). The workload was calculated twice; once using the measured average time of 7 minutes and 9 seconds (Table 1) and once using a minimal required time of 13 minutes 16 seconds (Table 2)

**Table 1:** Measured Workload for Primary TB Care for Each Patient in Pilot Study Area

	Overall	District 1	District 2	District 3
Overall average workload per TB patient (hrs)	**8,0**	**5,3**	**6,4**	**8,8**
Average workload clinical staff per Tb patient (hrs)	5,4	3,4	4,7	6,0
Average workload lab per patient (hrs)	2,7	1,9	1,8	2,8
New patients				
Average number visits New patient	51	18	36	69
Average workload clinical staff per New patient (hrs)	5,3	3,4	4,6	5,9
Average workload lab staff per New patient (hrs)	2,7	1,9	1,7	2,8
Average workload clinical and lab staff New pt (hrs)	**7,9**	**5,3**	**6,4**	**8,7**
Retreatment patients				
Average number visits Retreatment case	112	19	72	125
Average workload clinical staff Retr case (hrs)	7,9	3,5	6,2	8,3
Average workload lab staff per Retr case (hrs)	2,9	2,2	2,0	3,0
Average workload clinical and lab staff Retr case (hrs)	**10,8**	**5,6**	**8,2**	**11,4**

**Table 2:** Estimated Minimally Required Workload for Each Patient in Primary TB Care

	Overall	District 1	District 2	District 3
Overall average workload per TB patient (hrs)	**12,9**	**7,3**	**10,1**	**14,9**
Average workload clinical staff per Tb patient (hrs)	10,3	5,4	8,3	12,1
Average workload lab per patient (hrs)	2,7	1,9	1,8	2,8
New patients				
Average number visits New patient	51	18	36	69
Average workload clinical staff per New patient (hrs)	10,0	5,4	8,2	11,8
Average workload lab staff per New patient (hrs)	2,7	1,9	1,7	2,8
Average workload clinical and lab staff New pt (hrs)	**12,6**	**7,3**	**10,0**	**14,7**
Retreatment patients				
Average number visits Retreatment case	112	19	72	125
Average workload clinical staff Retr case (hrs)	17,0	5,5	12,4	18,3
Average workload lab staff per Retr case (hrs)	2,9	2,2	2,0	3,0
Average workload clinical and lab staff Retr case (hrs)	**19,9**	**7,7**	**14,4**	**21,3**

### Clinical service workload for TB treatment

A full treatment course for a new TB patient or a retreatment case is usually 6 or 8 months respectively and can be extended by one or two months if the sputum fails to clear from bacteria during the intensive phase (first 2 or 3 months) of treatment. Furthermore as indicated in Figure 1 the number of follow up visits during a complete tuberculosis treatment depends on the follow up protocol used in a country and may even vary by district, by provider and by patient. While some patients are required to come for daily observed treatment (usually 5 or 6 times a week) during the full 6 months of their treatment, others are observed daily only during the first 2 months of their treatment or are seen on a weekly, bi-weekly or monthly basis during their entire treatment.

The study used two sources to establish the average number of treatment follow up visits for each patient. First, patients currently under treatment were interviewed and asked the frequency with which they had to come for treatment. Second, the TB providers were interviewed and asked the frequency for each of their patients. The first method has the disadvantage of a selection bias. Patients that visit the clinic with higher frequency are overrepresented. The second method may lead to a re-call bias if the provider cannot refer to a register showing the frequency for each individual patient and/or a reporter bias if the standard protocol prescribes daily observed treatment by the provider, which is not in all situations feasible. We found that providers were rather well informed and seemed to be open in sharing reality and challenges faced, however they reported on average a 30% higher number of visits than the patients themselves. This means that both methods likely lead to overestimation. For that reason we use the lower number as reported by the patients for our calculations of the overall workload.

Our study showed no important differences in number of treatment visits for different forms of TB (smear positive, negative or extra-pulmonary TB). However important differences in frequency of visits were found among the three districts covered in the pilot study; an average of 18, 36 or 69 visits for new cases and 19, 72 and 125 visits for retreatment cases. Differences are influenced amongst others by distance to the clinics and by acceptance and use of community volunteers and family members as treatment observers.

Overall we found that 39% of new TB patients in the intensive phase of treatment reported to visit the clinic 5 days a week while 51% and 10% visited the clinic once a week and bi-weekly respectively (see Table 3). The frequency of visits diminished during the continuation phase of treatment with 28% still visiting daily while 31% visited weekly, 39% once every two weeks and 3% monthly. This leads to an overall average of 51 visits including adjustment for 3% patients that failed to sputum convert at month 2 and need an additional month of treatment. Retreatment cases (patients that had undergone earlier TB treatment) have not only a longer duration of treatment but also a higher average frequency of clinic visits and pay an average of 112 clinic visits.

**Table 3:** Number of Follow up Clinic Visits during Intensive and Continuation Treatment Phases for New and Retreatment TB cases

	New Patient intensive phase		New patient continuation phase		Retr Patient intensive phase		Retr Patient continuation phase	
	n	%	n	%	n	%	n	%
**All three district**								
5x/week	32	39%	22	28%	14	78%	11	55%
Weekly	41	51%	25	31%	3	17%	5	25%
Bi-weekly	8	10%	31	39%	1	6%	4	20%
Monthly	0	0%	2	3%	0	0%	0	0%
Overall	81	100%	80	100%	18	100%	20	100%
Average # visits (treatment phase)	21		30		50		62	
Average # visits (treatment duration)	51				112			

The actual duration of first time treatment consultation and follow up treatment consultations was measured by observation with a stopwatch and a check list. A TB treatment start up consultation lasted 12 minutes 41 sec (median 12 minutes 33 seconds, n = 19). Similar as in the diagnostic consultations we found that most first time consultations did not include all actions that should be done during a first time consultation such as full counselling, preparing a patient card, entering the patient in the register, prepare and provide drugs. In 7 of the in total 19 first time consultations that were observed the patient was counselled for at least 3 of 5 elements that are considered essential in TB treatment counselling, such as the diagnosis of TB, treatment process/duration, importance of adherence, explanation of the risk of HIV infection, offering of an HIV test. The consultation lasted on average 14 minutes 36 seconds. If also the use and role of a treatment supporter was discussed and a treatment card prepared, the consultation took on average 16 minutes 10 seconds (n = 3). This is considered the minimal duration of a first treatment consultation, but this time is still an underestimate as none of these patients were counselled for all 5 elements and the time for dispensing the drugs was not included in this measurement.

Follow up visits lasted on average 2 minutes and 34 seconds (median 2 minutes 50 sec, n = 199). The minimum required time if at least 4 most important tasks were done was 6 minutes 53 sec (n = 28).

### Laboratory service provision

The workload for the laboratory depends on the number of sputum smears to be performed to diagnose all TB cases and to monitor the treatment of smear positive TB cases. In our study area cultures are not done on a routine basis. The total time requirement for performance of sputum smears includes time for instructing a patient to produce good quality sputum, time to receive and label the sample, time to prepare and stain the smear and the time to read the slide and to record the result. Some of these were observed and clocked for individual patients (instruction and receiving sputum) while other activities are done in bulk for a number of smears (preparing, staining and reading slides). The latter activities are clocked for the total time and divided by the number of sputum samples. The time required to process one sputum sample is 12 minutes and 54 seconds on average (mean: 13 minutes and 9 seconds).

The calculation of the overall number of diagnostic sputum smears that is done for each identified patient is based on the total number of smears needed for diagnosis of all suspects. Suspect registers in the pilot study area were not well kept and there is no accurate record of the total number of smears done on suspects. We assume however that for each SS + case detected, 10 suspects were checked [37]. The current guidelines prescribe 2 sputum samples to be collected and checked for each suspect. This means that the estimated load of diagnostic smears is 20 times the SS + case notification.

For monitoring treatment effectiveness each SS + patient requires on average 2 follow up smears; one at month 2 (or month 3 for retreatment cases) and one at the end of treatment. In case of a positive smear at month 2 (or 3) this needs to be repeated one month later. With a smear conversion rate of 97% for Tanzania the total number of follow up smears is 2.03 per patient.

### Indirect time

The time spent on indirect TB related tasks includes administrative tasks, attending TB related trainings and meetings and receiving supervision. In addition we assessed for clinical staff the time spent on community outreach activities such as TB awareness raising, supervision of community volunteers and defaulter tracing.

For lab staff the indirect time includes also time spend on internal and external quality assurance. The total time spent annually on indirect tasks by clinical staff was divided by the total TB patient load in that clinic over the same year. The time spent on administration, training and supervision was 1.1 hour per patient. The time spent on community outreach was on average 0.6 hours per patient but showed major variations between districts. For laboratory personnel the indirect time spent in each facility was divided by the total number of sputum smears done in this clinic in a year to calculate an average time per smear resulting in 2 minutes to be added to the time per slide.

### Calculation of actual workload in TB

Actual TB-related workload at primary care level (T_Tb_) consists of:

1) Time needed for all TB related tasks performed by clinical staff dealing with TB patients (Tclin)

2) Time needed for all TB-related tasks by laboratory personnel (Tlab)

$$T_{\mathrm{TB}}=T_{\mathrm{clin}}+T_{\mathrm{lab}}$$

Additional file 1 gives a detailed description of the formula to calculate the overall workload on TB at primary care level within a given context on a year basis and explains the variables within the formula.

The times clocked showed a highly right skewed distribution. Therefore we calculated both mean and median times. For the calculation of workload we used the mean time as this results in a more accurate reflection of the total time needed including all patients. Subsequently this was used to calculate the average workload per patient.

Within the pilot study the current time spent to diagnose and treat one TB patient was calculated to be 8.0 hrs. Between the three districts included this varied from 5.3 to 8.8 hrs per patient (see Table 1). Variation depended mainly on the number of follow up treatment visits per patient which was highest in district 3. We found a difference in workload between new and retreatment patients of 7.9 against 10.8 hrs which can be explained by the greater number of follow up treatment consultations required.

### Assessment of quality and program performance

The measured average time per patient does not take into account the quality of the services provided. Using the time measured to perform consultations according to standard protocol one can calculate a 'minimum' time requirement per TB patient. In our pilot study the calculated 'minimum' time per patient was 12.6 hrs or 19.9 hrs for a newly diagnosed Tb patient and for a retreatment case respectively (see Table 2).

The influence of patient load and available staff time on the actual time spent per patient can be analysed by comparing average measured time in clinics with high and low patient load per staff available. Furthermore one could correlate time spent with programme performance by comparing treatment outcomes between districts with high and with low time spent per patient. In our pilot study we found signs of inefficient use of staff time in clinics with small number of patients, resulting in relative large amount of staff time spent on indirect tasks such as reporting and recording and other administrative tasks.

## Discussion

The pilot study clearly shows that within a disease such as Tuberculosis, that requires a relative long treatment and frequent patient provider contacts, the way the services are organised, the protocols/guidelines that are used and the extent to which standards are followed have an important influence on the workload of health providers.

There are a number of factors influencing the time required for a complete diagnosis and treatment of one TB patient. First this is influenced by the extent to which a health worker performs consultations according to the standard as described in the TB guidelines. In our example evidently health workers in most situations did not perform all tasks described in the standard guidelines. This resulted in an average diagnostic consultation duration of 7.2 minutes as opposed to a duration of 13.3 minutes for a consultation in which at least 3 out of 6 anamnesis questions were asked and some form of physical examination was performed. Similar differences were found in duration of treatment consultations.

A second factor influencing the time required per patient is the number of follow up treatment consultations that is prescribed by the provider. We found important differences in methods for service delivery (e.g. facility based DOT or community based DOT) in the different districts depending on the availability and acceptance of community level treatment supporters and remoteness of the area. Furthermore there seemed to be important variation in frequency of follow up visits between individual patients. It was not clear how decisions on frequency of follow up visits are made but these are likely to be influenced by patient's distance to the clinic and transport cost, and expectations on patient's ability to adhere to treatment including the risk of defaulting.

The methodology to establish TB related workload proposed here facilitates estimating the numbers of staff required to perform first line TB care. For this calculation a manager will need to take into account the number of staff available to perform the tasks, the percentage of their time they can spend on TB tasks. However, as a result of the large variation in the ways in which services are organized and the important influence of the different approaches on the time requirements it is important that the method for measuring workload takes all these factors into account.

Furthermore, given sub-national differences, one will need to calculate time estimates for each type of treatment protocol or programme design used in the country. If the aim is to extrapolate findings to national level workload estimates in order to calculate staff requirements, this needs to be followed by an assessment of the number of districts that use these different protocols. If the districts where data are collected are considered a good representation of the country, the average can be calculated from all data combined in these districts. If not, weighted calculations may be necessary.

Additionally, our workload assessment method provides a more accurate way in estimating the effects of change in protocols such as frequency of clinic visits on the overall workload of the health staff. If combined with programme performance data it can be used as a management tool to identify potential efficiency gains and to guide the debate on the cost efficiency of different potential programme decisions. The potential gains in improving the productivity of existing staff and system are crucial in the light of staff shortages [38].

For example, one striking finding was that out of the overall 8 hrs spent on each TB patient, 1.1 hrs was reportedly spent on indirect tasks such as administration, training and supervision. Given an indication that clinics with lower numbers of TB patients may spend disproportionate amounts of time on these tasks, this may warrant a search for alternative approaches for reporting practices.

Other potential benefits of using this workload assessment are the possibility to combine measurements of actual workload with a study on perceived workload amongst the same health workers. It is possible to find a low correlation between actual and perceived work load, for instance in a situation where multiple tasks in different departments can lead to a perceived busy work schedule, but not necessarily a productive use of time. In our interviews, health workers and managers complained about important staff shortages, leading to multi-tasking and perceptions of a heavy workload. Workload that is perceived as heavy may lead to demotivated staff or burn out [39].

As in any method there are limitations to the level of accuracy and validity. The most important limitation we identified is the difficulty to estimate the amount of staff time spent on indirect TB related tasks and on their absence. In our protocol we base this on standardised interviews with managers in the clinics. The reported time spent on recording and reporting, stock management, TB meetings, receiving supervision and attending TB trainings varied enormously between the different interviewees. Scatter plots of time per patient against patient load do not show a clear pattern, but especially in clinics with low numbers of TB patients we identified major variations in indirect time spent per TB patient.

Similar variations were found in time that was spent on community outreach. While some staff reported substantive outreach activities on a weekly basis, others hardly ever left the clinic.

The described methodology excludes time spent on care for hospitalised patients, which is no problem for Tanzania where the number of patients hospitalized for TB is extremely limited. The study protocol will need to be adapted in situations where hospitalisation is more common. Other important time requirements such as tasks performed by TB managers or time required for the management of multi drug resistant TB also need to be estimated separately.

## Conclusions

We present a newly designed methodology to measure workload related to primary level TB care within a specific context and programme design that answers to a widely felt need to estimate staff requirements in TB care.

Piloting of the methodology provided evidence that it is feasible to be used under field circumstances both in urban and rural settings. The outcome of the assessment of time requirements allows not only estimating staff requirements, it also differentiates the amounts of time spent on different tasks within TB care. The method therefore can be used to identify those tasks that staff spent most of their time on. Furthermore the protocol assesses a number of variables such as the duration of consultations, frequency of follow up visits of patients under treatment, patient flow, and the number of consultations before a diagnosis is made. In this way it can be used to identify areas for potential efficiency gains through changes in program design, patient flows and frequency or organization of patient follow up. The observation of completeness of tasks performed allows correlation of time required for specific tasks to quality according to guidelines.

The method we have used will need to be further tested in other countries to be refined and to translate it into a management tool so urgently required for rational planning of HRH in TB control programs.

## Competing interests

The authors declare that they have no competing interests.

## Authors' contributions

LB was one of the main developers of the study methodology, participated in the analysis of the results of the pilot study, and drafted the manuscript. SvdH and MD participated in the development of the study methodology and the analysis of the results of the pilot study and contributed to the drafting of the manuscript. SM was overseeing the implementation of the pilot study. AK and EN took part in data collection and analysis of the results. LO contributed to the analysis of results. All authors have read and approved the final manuscript.

## Supplementary Material

Additional file 1 **Formula to Calculate TB related Workload for Clinical and Laboratory Staff within a given Setting**. Click here for file
