# Supplementary material for: Measuring workload for tuberculosis service provision at primary care level: a methodology
Source: Hum Resour Health. 2012 May 28;10:11. doi: 10.1186/1478-4491-10-11 (PMC3375184; doi:10.1186/1478-4491-10-11)
Supplement: Additional file 1 — Formula to Calculate TB related Workload for Clinical and Laboratory Staff within a given Setting. [file 1478-4491-10-11-S1.PDF]

Total workload $T_{TB} = T_{clin} + T_{lab}$

T_{TB}	=	Total workload: clinical and laboratory service time required for diagnosis, treatment and follow-up of all tuberculosis (TB) cases by year
T_{clin}	=	Clinical Workload: clinical service time required for treatment and follow-up of all diagnosed TB cases by year
T_{lab}	=	Laboratory Workload: laboratory service time required for diagnosis and follow-up of all TB cases

1. Clinical workload $T_{clin} = T_{clin\text{-}Dx} + T_{clin\text{-}Tx} + T_{clin\text{-}Indirect}$

$T_{clin\text{-}Dx}$	=	Clinical diagnostic workload: service time required for diagnosis of all TB cases in a year
$T_{clin\text{-}Tx}$	=	Clinical treatment workload: service time required for treatment and follow-up of all diagnosed TB cases in a year
$T_{clin\text{-}Indirect}$	=	Clinical indirect workload: Average time spent by HW's on a year basis on Tb related administration, meetings, training, supervision, recording & reporting and community outreach

Clinical diagnostic workload $T_{clin\text{-}Dx} = T_{clin\text{-}Dx\text{-}cons} * N_{Dx\text{-}cons/susp} * N_{Susp}$

$T_{clin\text{-}Dx\text{-}cons}$	=	Clinical service time required for a diagnostic consultation of a patient with complaints consistent with TB
$N_{Dx\text{-}cons/susp}$	=	Average number of diagnostic consultations needed to reach a diagnosis for patient with complaints consistent with TB
N_{Susp}	=	Total number TB suspect cases seen in a district during a full year

Clinical treatment workload $T_{clin\text{-}Tx} = T_{clin\text{-}Tx\text{-}new} + T_{clin\text{-}Tx\text{-}retr}$

$$T_{clin\text{-}Tx\text{-}new} = (T_{Tx\text{-}cons\text{-}1} + (T_{Tx\text{-}cons\text{-}fu} * N_{Tx\text{-}cons\text{-}fu/new})) * N_{new}$$

$$T_{clin\text{-}Tx\text{-}retr} = (T_{Tx\text{-}cons\text{-}1} + (T_{Tx\text{-}cons\text{-}fu} * N_{Tx\text{-}cons\text{-}fu/retr})) * N_{retr\backslash}$$

$T_{clin\text{-}Tx\text{-}new}$	=	Time required treating and following up all new TB cases
$T_{clin\text{-}Tx\text{-}retr}$	=	Time required treating and following up all retreatment TB cases
$T_{Tx\text{-}cons\text{-}1}$	=	Average time spent on 1st treatment consultation of a TB case
$T_{Tx\text{-}cons\text{-}fu}$	=	Average time spent on follow up consultation of a TB case
$N_{Tx\text{-}cons\text{-}fu/new}$	=	Average number of follow up consultations for a new TB case
$N_{Tx\text{-}cons\text{-}fu/retr}$	=	Average number of follow up consultations for a TB retreatment case
N_{new}	=	Total number of new TB cases during a full year
N_{retr}	=	Total number of retreatment TB cases during a full year

2 Laboratory workload $T_{lab} = T_{lab\text{-}Dx} + T_{lab\text{-}Tx} + T_{lab\text{-}Indirect}$

$T_{lab\text{-}Dx}$	=	Laboratory diagnostic workload: service time required for diagnosis of all TB cases in a year
$T_{lab\text{-}Tx}$	=	Laboratory treatment workload: service time required for follow-up of all diagnosed TB cases during treatment in a year
$T_{lab\text{-}Indirect}$	=	Laboratory indirect workload: Average time spent by laboratory personnel on a year basis on TB related administration, meetings, supervision, training, recording & reporting and quality assurance

Laboratory diagnostic workload $T_{lab\text{-}Dx} = T_{sm} * N_{Susp} * N_{Dx\text{-}sm}$

T_{sm}	=	Average time spent on a performing one sputum
N_{Susp}	=	Total number TB suspect cases seen during a full year
$N_{Dx\text{-}sm}$	=	Number of diagnostic smears performed per TB suspect (here n=2)

Laboratory treatment workload $T_{lab\text{-}Tx} = T_{lab\text{-}Tx\text{-}new} + T_{lab\text{-}Tx\text{-}retr}$

$$T_{lab\text{-}Tx\text{-}new} = T_{Sm} * N_{Tx\text{-}sm/newpat} * N_{New}$$

$$T_{lab\text{-}Tx\text{-}retr} = T_{Sm} * N_{Tx\text{-}Sm/Retrpat} * N_{Retr}$$

$T_{lab\text{-}Tx\text{-}new}$	=	Time required to perform smears for treatment follow up of all diagnosed new TB cases
$T_{lab\text{-}Tx\text{-}retr}$	=	Time required to perform smears for treatment follow up of all retreatment TB cases
$N_{Tx\text{-}sm/new}$	=	Average number of follow up smears for a new TB case
N_{Newsm+}	=	Total number of new, sputum smear positive TB cases during a year
$N_{Tx\text{-}sm/retr}$	=	Average number of follow up smears for a retreatment TB case
$N_{Retrsm+}$	=	Total number of retreatment, sputum smear positive TB cases during a year
